# Bacteriome Signature in SARS-CoV-2-Infected Patients Correlates with Increased Gut Permeability and Systemic Inflammatory Cytokines

**DOI:** 10.3390/microorganisms13061407

**Published:** 2025-06-16

**Authors:** Larissa S. Souza, Alexandre S. Ferreira-Junior, Pedro C. Estella, Ricardo K. Noda, Lhorena F. Sousa, Miguel T. Y. Murata, Lucas A. L. Carvalho, João L. Brisotti, Daniel G. Pinheiro, Josias Rodrigues, Carlos M. C. B. Fortaleza, Gislane L. V. de Oliveira

**Affiliations:** 1 Department of Genetics, Microbiology and Immunology, Institute of Biosciences (IBB), Sao Paulo State University (UNESP), Botucatu 18618-681, Brazil; larissa.silva-souza@unesp.br (L.S.S.); alexandre.soares@unesp.br (A.S.F.-J.); josias.rodrigues@unesp.br (J.R.); 2 Botucatu School of Medicine (FMB), Sao Paulo State University (UNESP), Botucatu 18618-687, Brazil; pedro.coltro@unesp.br (P.C.E.); kazumi.noda@unesp.br (R.K.N.); carlos.fortaleza@unesp.br (C.M.C.B.F.); 3 Santa Casa Hospital, Ribeirão Preto 14085-000, Brazil; dralhorenaferreira@gmail.com (L.F.S.); miguelmurata@gmail.com (M.T.Y.M.); jlbrisotti@gmail.com (J.L.B.); 4 Department of Agricultural, Livestock and Environmental Biotechnology, School of Agricultural and Veterinary Sciences (FCAV), Sao Paulo State University (UNESP), Jaboticabal 14884-900, Brazil; lucas.amoroso@unesp.br (L.A.L.C.); daniel.pinheiro@unesp.br (D.G.P.)

**Keywords:** COVID-19, post-COVID-19 condition, gut-lung axis, bacteriome profile, inflammatory cytokines, gut permeability

## Abstract

The COVID-19 pandemic has highlighted the complex interplay between the gut microbiota and systemic immune responses, particularly through the gut–lung axis. Disruptions in gut microbial diversity and function—commonly referred to as dysbiosis—have been increasingly implicated in the pathogenesis of SARS-CoV-2 infection. In this study, we assessed the gut bacteriome and permeability in SARS-CoV-2-infected patients using 16S sequencing and ELISA assays, respectively. We also measured blood inflammatory cytokines and fecal secretory IgA to evaluate systemic and mucosal immune responses. Significant alterations in both alpha and beta diversity metrics were observed in patients with COVID-19 (*n* = 79) and those with post-COVID-19 condition (*n* = 141) compared to the controls (*n* = 97). Differential abundance and taxonomic analyses revealed distinct microbial profiles in the infected groups. Increased plasma levels of IL-2, IL-6, IL-17A, IFN-γ, and zonulin were detected in patient samples. Some genera were elevated during acute infection, which was positively correlated with C-reactive protein, while *Enterobacteriaceae* and *Escherichia-Shigella* were associated with increased zonulin levels, indicating compromised intestinal barrier function. These findings suggest that gut dysbiosis may contribute to bacterial translocation and systemic inflammation. Overall, our results highlight the importance of the gut–lung axis and suggest that modulating the gut microbiota could support immune regulation in SARS-CoV-2 infection.

## 1. Introduction

Coronavirus Disease 2019 (COVID-19) is an infectious disease caused by Severe Acute Respiratory Syndrome Coronavirus 2 (SARS-CoV-2). The virus spread rapidly across the globe and was officially declared a global pandemic by the World Health Organization (WHO) on 11 March 2020 [1,2]. Since then, SARS-CoV-2 has infected over 777 million individuals worldwide, resulting in more than 7 million deaths and causing unprecedented disruptions in healthcare systems, economies, and daily life [3]. Among the most severely affected countries, Brazil reported more than 39 million confirmed cases and 716,238 deaths [4].

Clinical manifestations of COVID-19 range from mild respiratory symptoms to severe acute respiratory distress syndrome (ARDS) and multiple organ failure (MOF), especially in vulnerable populations such as the elderly and individuals with underlying health conditions [5,6,7,8]. Furthermore, many patients develop persistent symptoms following the resolution of the acute illness—a condition recognized as post COVID-19 condition (PCC) [9,10,11]. PCC may last beyond four weeks post-infection and persist for months or even years, involving a spectrum of physical, cognitive, and psychological impairments [12,13]. Global estimates suggest that PCC affects between 6% and 50% of COVID-19 survivors, impacting over 65 million people worldwide [11,14,15,16].

The progression of COVID-19 to severe forms has been closely linked to immune system dysregulation, particularly a hyperinflammatory response marked by the excessive release of cytokines [17,18,19]. This immunopathogenesis involves delayed type I interferon responses, inflammasome activation, the extrusion of neutrophil extracellular traps in pulmonary tissues, and PANoptosis triggered by the synergistic action of TNF and IFN-γ [20,21]. Additionally, the viral envelope engagement of TLR-2 and downstream cytokine cascades contribute to the inflammatory milieu [22]. Inflammatory monocytes and macrophages produce high levels of IL-6 and other cytokines, fueling the so-called “cytokine storm” [23]. Meanwhile, CD4^+^ Th1 cells exhibit signs of exhaustion, and CD8^+^ cytotoxic T cells undergo apoptosis, compromising the clearance of viral reservoirs [20]. Furthermore, TNF and IFN-γ contribute to the depletion of germinal centers in lymphoid tissues, leading to lymphopenia and impaired antibody production [20,24].

SARS-CoV-2 infection not only causes acute immune disruption but may also lead to a chronic inflammatory state in PCC patients [10,25]. Studies have reported a sustained increase in inflammatory cytokines (e.g., IFNs and IL-6) and the presence of autoantibodies for months post-infection [25,26]. Recovery from PCC symptoms appear to correlate with the gradual restoration of immune function within approximately two years following the initial infection [27].

Host immunocompetence significantly influences the clinical and immunological response to SARS-CoV-2, and studies suggest that this response is modulated by the intestinal microbiota [28]. A bidirectional relationship—referred to as the gut–lung axis—exists between the gastrointestinal and respiratory mucosa and plays a crucial role in determining immune outcomes in both health and disease [29,30,31,32]. The gut bacteriome, a central player in this axis, affects local and systemic immune regulation. Disruption of the gut microbiota has been implicated in the pathogenesis of respiratory diseases such as asthma, chronic obstructive pulmonary disease, pneumonia, and COVID-19 [30,31,32,33,34,35,36]. Moreover, dysbiosis may impair intestinal barrier integrity, promoting increased gut permeability and bacterial translocation into the bloodstream, thereby contributing to systemic inflammation and worsening disease outcomes and COVID-19 severity [37,38,39].

Despite emerging evidence supporting the role of the gut–lung axis in respiratory health [29,30,31,32,33,34,35,36], studies evaluating gut microbiota alterations during COVID-19 in the Brazilian population remain lacking. Moreover, the potential link between dysbiosis, gut permeability, and systemic inflammation in this context has not yet been explored. Therefore, the aim of this study was to evaluate alterations in the gut bacteriome of SARS-CoV-2-infected patients and investigate their correlation with intestinal permeability and systemic inflammatory markers. Unraveling the mechanisms underlying the gut–lung axis may offer novel strategies for the prevention and treatment of respiratory complications and post-COVID-19 sequelae.

## 2. Patients and Methods

### 2.1. Study Design, Ethical Aspects and Patients’ Enrollment

This observational study was performed in accordance with the Declaration of Helsinki and was approved by the Research Ethics Committee from Sao Paulo State University (process number 4,310,336/2020). All participants, over 18 years of age, signed an informed consent form, peripheral blood samples were collected, and fecal samples were requested and delivered within 5 days. The participants included in this study (*n* = 317) were enrolled between October 2020 and December 2021 in two different hospitals in Brazil (Ribeirao Preto Santa Casa and Botucatu Clinical Hospital), in addition to Sao Jose do Rio Preto Institute of Hematology Laboratory.

A total of 220 unvaccinated patients infected with SARS-CoV-2 were included, confirmed by RT-qPCR testing of oropharyngeal or nasopharyngeal swabs. Patients with moderate-to-severe COVID-19 were excluded if their hospitalizations were due to conditions unrelated to COVID-19 complications. Patients were classified into three severity groups based on clinical presentation: mild (home isolation and recovery with symptoms such as fever, cough, sore throat, headache, fatigue, or loss of taste or smell); moderate (evidence of lower respiratory tract involvement with oxygen saturation < 94%, managed with non-invasive oxygen therapy); and severe (requiring advanced respiratory support including invasive oxygen therapy, ICU admission, or mechanical ventilation) [6,7]. Demographic, clinical, and laboratory data were collected, encompassing gender, age, height, weight, body mass index (BMI), disease severity, symptoms, sequelae, comorbidities, medications, hospitalizations, chest radiographs, and C-reactive protein levels.

The control group comprised 97 individuals without COVID-19 or any underlying medical conditions. Additional exclusion criteria included the use of anti-inflammatory drugs, immunosuppressants, antibiotics, or vaccination within the past 30 days, as well as chronic diarrhea.

### 2.2. Bacteriome Characterization by 16S Sequencing

DNA was obtained from 200 mg of fecal samples by using the QIAamp Fast DNA Stool Mini Kit (Qiagen, CA, USA), according to the manufacturer’s protocol. The analysis of the bacteriome was based on the sequencing of 16SV6 rDNA amplicons in the Ion Torrent Personal Genome Machine™, following a clonal amplification (emulsion PCR). Pooled barcoded amplicons were attached to the surface of ion sphere particles (ISPs) using the IonPGM™ Template OT2 400 kit and the corresponding protocol. Emulsion PCR was carried out in the Ion OneTouch™ 2 System. After amplification quality checking, ISP enrichment was performed in the Ion OneTouch™ Enrichment System (Thermo Fisher Scientific, Waltham, MA, USA). Sequencing primers were then annealed to the ISPs’ single-stranded DNA, following the Ion PGM sequencing 400 kit protocol. Low-quality and polyclonal sequence reads, as well as primers and barcodes, were filtered out, and the 16SV6 rDNA sequences were available as a FastQ file. The raw data was deposited in the NCBI Sequence Read Archive (SRA) under BioProject PRJNA1189098.

### 2.3. Zonulin and Secretory Immunoglobulin a Quantification by ELISA Assays

Approximately 5 mL of peripheral blood was collected from participants in an EDTA k2 tube, and the plasma was separated by centrifugation at 1372× *g* for 10 min at 4 °C. Plasma zonulin measurements were carried out by using the Human Zonulin ELISA Kit (Elabscience, Bethesda, MD, USA), according to the manufacturer’s recommendations. Fecal secretory immunoglobulin A (sIgA) was quantified by the commercial Human IgA ELISA kit (Elabscience, Bethesda, MD, USA), according to the manufacturer’s protocol. For both ELISA assays, the optical density (OD) was read at 450 nm in a spectrophotometer. The calibration curves were constructed in Excel spreadsheets using the formula y = ax + b, where x and y were two dependent variables (OD and concentration). The concentrations were calculated by converting the OD into ng/mL.

### 2.4. Cytokine Quantification by Cytometric Bead Array and ELISA Assay

Approximately 5 mL of peripheral blood was collected from participants in an EDTA k2 tube, and the plasma was separated by centrifugation at 1372× *g* for 10 min at 4 °C. Plasma samples were used for cytokine quantification by using a cytometric bead array (Human Th1/Th2/Th17 Kit, BD Biosciences, San Jose, CA, USA). The levels of interleukin (IL)-2, IL-4, IL-6, IL-10, IL-17A, interferon-gamma (IFN-γ), and tumor necrosis factor (TNF) were detected by a flow cytometer (FACSCanto™ II, BD Biosciences, Franklin Lakes, NJ, USA). The results were analyzed with BDFCAP array™ v3 software and were expressed in pg/mL. The Transforming Growth Factor-β (TGF-β) cytokine was measured by the TGF-β1 sandwich-ELISA Kit (Elabscience, Bethesda, MD, USA), according to the manufacturer’s protocol. The absorbance was read at 450 nm, and the results were presented as ng/mL.

### 2.5. Bioinformatic and Statistical Analyses

The sequence initial quality was assessed by using the FastQC program (v.0.11.9) [40]. The reads were then submitted to the DADA2 pipeline (v.1.22.0) [41] using the corresponding package in the R statistical program (v.4.1.2) (R Core Team, 2023). The quality control steps included size truncation (truncLen = 250), leading trimming removal (trimLeft = 10) and quality filtering (maxEE = 2) by the “filterAndTrim” function. Amplicon variant sequences (ASVs) were identified for each sample, and possible chimeric sequences were filtered using the “removeBimeraDenovo” function. Taxonomic classification was performed by using the “assignTaxonomy” function, using the RDP reference database (v.18) [42]. The ASVs were also aligned with BLAST Plus against the NCBI RefSeq 16S rRNA database [43].

The phylogenetic relationship between ASVs was established with the Neighbor-Joining algorithm using the “NJ” function and statistically validated by the bootstrap method with the “bootstrap.pml” function, both from the R package “phangorn” (v.2.10.0) [44]. The counts, taxonomic annotations and the phylogenetic tree were exported in the “phyloseq” format (R package “phyloseq”) (v.1.38.0) [45]. The phyloseq object was transformed into compositional data by the “phyloseq_standardize_otu_abundance” function from the “metagMisc” package (v.0.04) for subsequent analyses [46].

Sequencing coverage was assessed using rarefaction curves generated using the “amp_rarecurve” function from the “ampvis2” package (v.2.7.17) [47]. For alpha diversity, observed richness and diversity indices (Shannon, Gini–Simpson, and Faith’s phylogenetic diversity) were estimated using the “alpha” function from the “microbiome” package (v.1.16.0) [48]. Beta diversity was analyzed using the Bray–Curtis, Jaccard, and UniFrac (weighted and unweighted) dissimilarity indices obtained using the “distance” function from the “phyloseq” package. The dispersion of samples within each group was also assessed.

The alpha diversity metrics, distance dispersion, relative abundances, cytokines, zonulin, and SIgA were compared using the Kruskal–Wallis test (*p* < 0.05). When the data showed significance, the means were compared with the Wilcoxon post hoc test paired at a 5% probability (*p* < 0.05). To assess the differences in beta diversity between the groups, Permutational Multivariate Analysis of Variance (PERMANOVA) was used using the “adonis2” function of the “vegan” package (v.2.6.4) [49]. The post hoc analysis was performed with the “pairwise.adonis” function (R package “pairwiseAdonis”) (v.0.4) [50]. The multidimensional distances were ordered by Principal Coordinate Analysis (PCoA). The graphical representations of the analyses were generated in the “R” program using the “ggplot2” package (v.3.5.1) [51]. All results regarding cytokine, zonulin, and sIgA levels are expressed as mean ± standard error of the mean (SEM).

## 3. Results

### 3.1. Clinical and Demographic Characteristics of SARS-CoV-2 Infected Patients

A total of 220 patients infected with SARS-CoV-2 were included in the analysis, of which 79 were in the acute phase of the disease and 141 had post-COVID-19 condition (PCC). In the COVID-19 group, 58% of patients were female and 42% were male, with a mean age of 52 years, and the patients’ condition was classified as mild (*n* = 28), moderate (*n* = 41), or severe (*n* = 10). In the PCC group, 62% of the patients were female, and 38% were male, with a mean age of 41 years, and the disease was classified as mild (*n* = 114), moderate (*n* = 13), or severe (*n* = 14). The control group (CTL) was 85% female and 15% male, with a mean age of 44 years. The C-reactive protein (CRP) concentrations were significantly higher in those with COVID-19 when compared with the PCC patients (*p* < 0.001). Table 1 summarizes the clinical and demographic details of the SARS-CoV-2-infected patients and control subjects.

### 3.2. Bacteriome Signature in Patients Infected with SARS-CoV-2 Virus

**Figure 1:**
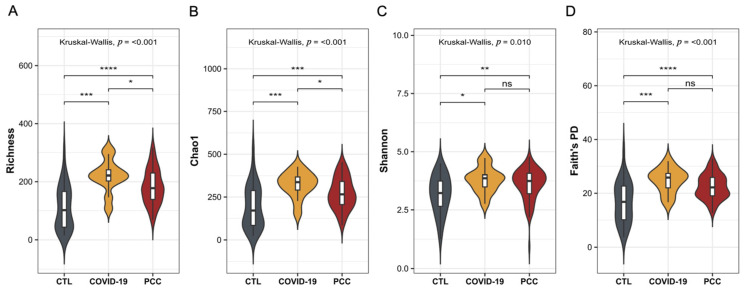
Alpha diversity analysis of the gut bacteriome from control subjects (CTL), COVID-19 patients, and post-COVID-19 condition (PCC) patients. (**A**) Richness observed; (**B**) Chao1 index; (**C**) Shannon’s diversity; (**D**) Faith’s phylogenetic diversity. ****: *p* < 0.0001; ***: *p* < 0.001; **: *p* < 0.01; *: *p* < 0.05; ns: *p* ≥ 0.05.

**Figure 2:**
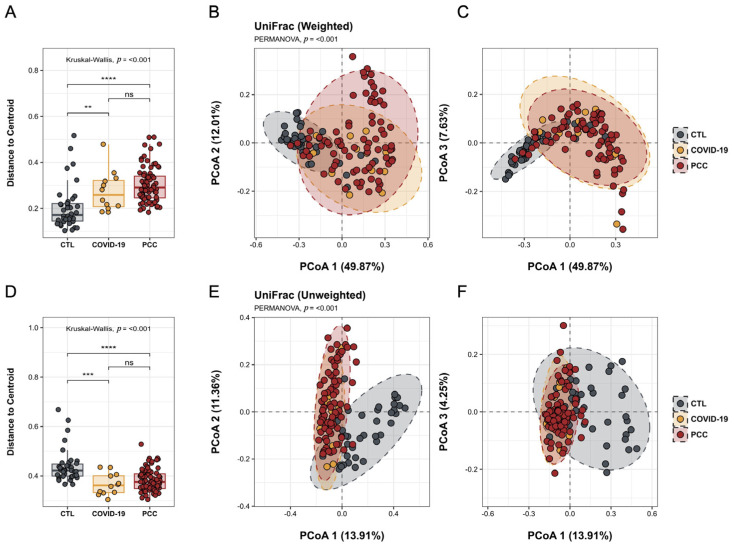
Beta diversity analysis of the gut bacteriome from control subjects (CTL), acute COVID-19 patients, and post-COVID-19 condition (PCC) patients using weighted and unweighted UniFrac metrics. (**A**,**D**) Distances to centroid; (**B**,**C**,**E**,**F**) principal coordinate analysis with different clusters. ****: *p* < 0.0001; ***: *p* < 0.001; **: *p* < 0.01; ns: *p* ≥ 0.05.

To investigate significant changes in the gut bacteriome of patients infected with SARS-CoV-2, we performed 16S sequencing and evaluated the alpha and beta diversities. For alpha diversity, the observed richness and diversity indices were estimated. We observed significant differences (*p* < 0.001) in richness, Chao1, Shannon’s (*p* = 0.010), and Faith’s phylogenetic diversity metrics in the patients’ samples (COVID-19 and PCC) when compared with the CTL group (Figure 1A–D). We also detected significant differences (*p* < 0.001) in the microbial communities found in the COVID-19 and PCC patients, compared with the CTL group, with different clusters in the principal coordinate analysis (PCoA) (Figure 2A–F).

**Figure 3:**
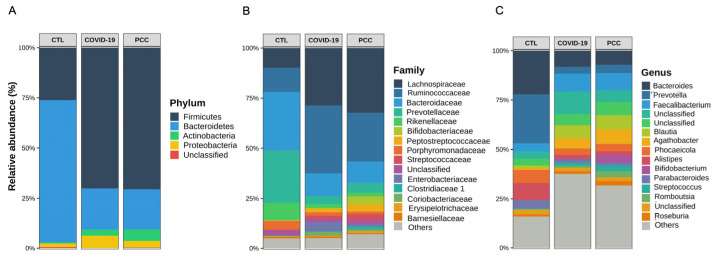

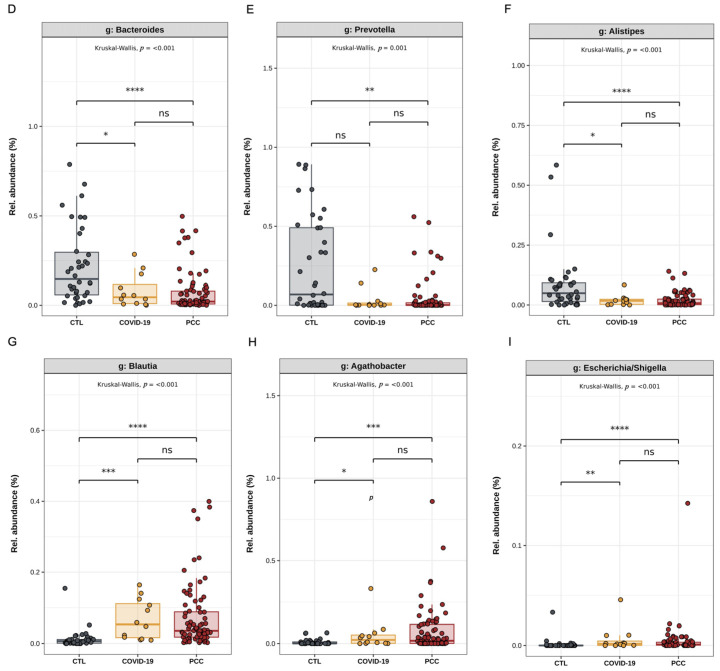
Taxonomical distribution and differential abundance analysis of the gut bacteriome from control subjects (CTL), acute COVID-19 patients, and post-COVID-19 condition (PCC) patients. (**A**–**C**) Taxa abundances; (**D**–**I**) genus differential abundance analysis. ****: *p* < 0.0001; ***: *p* < 0.001; **: *p* < 0.01; *: *p* < 0.05; ns: *p* ≥ 0.05.

Regarding the differential abundance analysis and taxonomic distribution, we observed significant differences (*p* < 0.001) among the COVID-19, PCC, and CTL groups in terms of Bacteroidota (formerly Bacteroidetes) (22.4% vs. 21.9% vs. 73.3%) and Bacillota phyla (formerly Firmicutes) (67.9% vs. 68.6% vs. 24.2%) (Figure 3A). Decreased *Prevotellaceae* (4.4% vs. 5.1% vs. 31.2%) and *Bacteroidaceae* (12.3% vs. 11.7% vs. 28.7%) abundances were detected in the COVID-19 and PCC groups, as well as increased *Ruminococcaceae* (31.5% vs. 24.9% vs. 12.2%) and *Lachnospiraceae* (28.8% vs. 30.4% vs. 8.9%), compared with the CTL group. In addition, the *Enterobacteriaceae* family was also significantly increased (*p* = 0.013) in the COVID-19 group (4.6% vs. controls 0.28%) (Figure 3B). Finally, the *Bacteroides* (8.9% vs. 7.5% vs. 21.6%)*, Prevotella* (3.7% vs. 4.5% vs. 29.7%), and *Alistipes* genera (2.0% vs. 1.8% vs. 5.4%) were significantly decreased in the COVID-19 and PCC groups and *Blautia* (7.1% vs. 6.4% vs. 0.71%), *Agathobacter* (4.1% vs. 7.1% vs. 0.85%), and *Escherichia-Shigella* (0.64% vs. 0.50% vs. 0.19%) were increased compared with the CTL group (Figure 3C–I).

### 3.3. Increased Gut Permeability in SARS-CoV-2-Infected Patients

**Figure 4:**
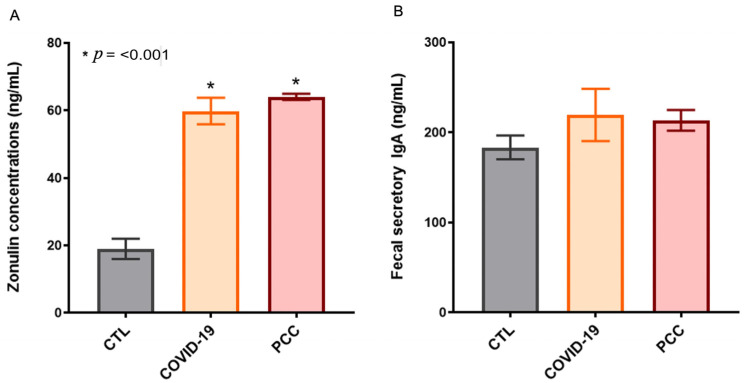
Zonulin plasma concentrations (**A**), and fecal secretory IgA (**B**) in control subjects (CTL), acute COVID-19 patients, and post-COVID-19 condition (PCC) patients.

Since we detected significant changes in the intestinal bacteriome in patients infected with SARS-CoV-2, we also checked the integrity of the gastrointestinal barrier by measuring plasma zonulin levels and fecal secretory IgA. We observed a significant increase (*p* < 0.001) in zonulin concentrations in COVID-19 (60 ± 3.9 ng/mL) and PCC patients (64 ± 0.9 ng/mL) when compared with the CTL group (19 ± 3 ng/mL) (Figure 4A). The secretory IgA concentrations in fecal samples from COVID-19 (220 ± 29 ng/mL) and PCC (214 ± 11 ng/mL) patients did not differ from the CTL group (184 ± 13 ng/mL) (Figure 4B).

### 3.4. Increased Inflammatory Cytokines in SARS-CoV-2-Infected Patients

**Figure 5:**
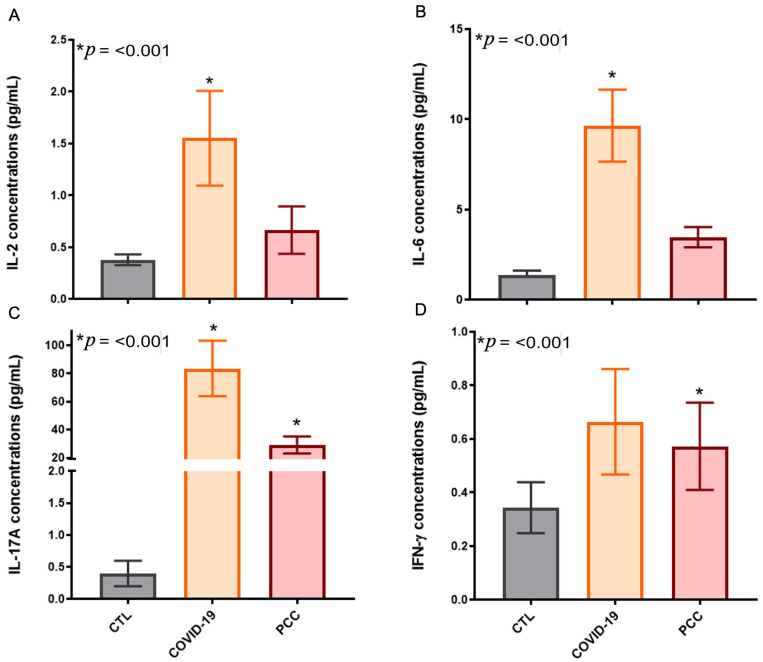
Cytokine concentrations in control subjects (CTL), COVID-19 patients, and post-COVID-19 condition (PCC) patients. (**A**) IL-2 plasma levels, (**B**) IL-6, (**C**) IL-17A, and (**D**) IFN-γ.

In order to verify the systemic immunity in blood plasma samples, we quantified the IL-2, IL-4, IL-6, IL-10, IL-17A, IFN-γ, TGF-β, and TNF concentrations in patients and healthy individuals. We detected significant differences in IL-2, IL-6, IL-17A, and IFN-γ in COVID-19 (IL-2: 1.6 ± 0.46 pg/mL, IL-6: 9.7 ± 2.0, IL-17A: 84 ± 20, IFN-γ: 0.66 ± 0.20) and PCC (IL-2: 0.67 ± 0.23 pg/mL, IL-6: 3.5 ± 0.56, IL-17A: 29 ± 6.0, IFN-γ: 0.57 ± 0.16) patients when compared to the CTL group (IL-2: 0.38 ± 0.05 pg/mL, IL-6: 1.4 ± 0.25, IL-17A: 0.40 ± 0.20, IFN-γ: 0.34 ± 0.09) (Figure 5). No differences were found in IL-4, IL-10, TGF-β, and TNF concentrations between the SARS-CoV-2-infected patients and the controls.

### 3.5. Correlations Among the Bacteriome, Clinical Data, Gut Permeability, and Cytokines

**Figure 6:**
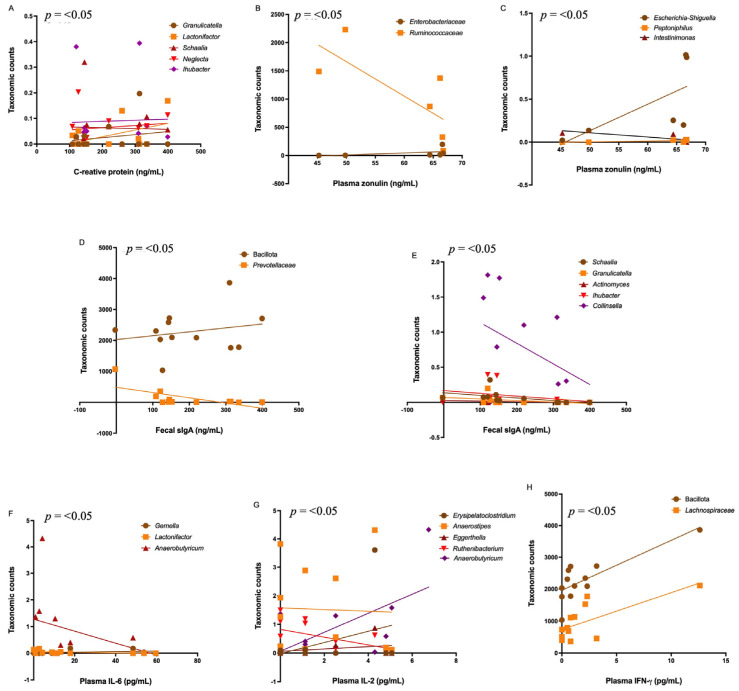
Correlations between taxonomic counts of the gut bacteriome and clinical and laboratory data in COVID-19 patients. (**A**) C-reactive protein with differentially increased genera. (**B**,**C**) Plasma zonulin levels with differentially increased families and genera. (**D**,**E**) Fecal sIgA levels with differentially increased Bacillota, *Prevotellacea,* and some genera. (**F**–**H**) Plasma concentrations of IL-6, IL-2 and IFN-γ with differentially increased taxa.

Since we found significant differences in the gut bacteriome in SARS-CoV-2-infected patients, we investigated moderate-strong correlations between the bacterial taxonomic counts and clinical data. No moderate-strong correlations between the bacterial taxonomic counts and clinical results in PCC patients were found. On the other hand, some differentially increased genera in COVID-19 patients correlated with C-reactive protein levels, including *Schaalia* (r = −0.87; *p* < 0.001), *Ihubacter* (r = −0.85; *p* < 0.001), *Lactonifactor* (r = 0.78; *p* = 0.004), *Neglecta* (r = 0.67; *p* = 0.014), and *Granulicatella* (r = −0.54; *p* = 0.046). (Figure 6A).

Regarding gut permeability, some taxa differentially increased in COVID-19 patients and were correlated with zonulin concentrations, including *Enterobacteriaceae* (r = 0.94; *p* = 0.008), *Ruminococcaceae* (r = −0.88; *p* = 0.016), *Escherichia-Shigella* (r = 0.88; *p* = 0.016), *Peptoniphilus* (r = 0.77; *p* = 0.05), and *Intestinimonas* (r = −0.92; *p* = 0.011) (Figure 6B,C). Although we found no differences in fecal secretory IgA levels between the patients and the controls, we observed some moderate–strong inverse correlations with some differentially increased taxa in COVID-19, including Bacillota (formerly Firmicutes) (r = 0.54; *p* = 0.035), *Prevotellaceae* (r = −0.62; *p* = 0.017), *Schaalia* (r = −0.83; *p* < 0.001), *Granulicatella* (r = −0.65; *p* = 0.013), *Actinomyces* (r = −0.55; *p* = 0.035), *Ihubacter* (r = −0.53; *p* = 0.038), and *Collinsella* (r = −0.51; *p* = 0.045). Also, we observed a moderate–strong positive correlation between IgA levels and the Bacillota phylum (r = 0.54; *p* = 0.035) and *Lactonifactor* genera (r = 0.79; *p* = 0.002) (Figure 6D,E).

Some differentially expressed genera in COVID-19 patients correlated with inflammatory IL-6 concentrations, including *Gemella* (r = 0.58; *p* = 0.025), *Lactonifactor* (r = −0.58; *p* = 0.025), and *Anaerobutyricum* (r = −0.52; *p* = 0.042) (Figure 6F). Similarly, some genera correlated with IL-2 concentrations, such as *Erysipelatoclostridium* (r = 0.77; *p* = 0.003), *Anaerostipes* (r = 0.58; *p* = 0.025), *Eggerthella* (r = 0.56; *p* = 0.032), *Ruthenibacterium* (r = 0.55; *p* = 0.032), and *Anaerobutyricum* (r = 0.52; *p* = 0.042) (Figure 6G). Also, IFN-γ levels correlated with taxonomic counts of Bacillota (formerly Firmicutes) (r = 0.70; *p* = 0.006) and *Lachnospiraceae* (r = 0.55; *p* = 0.034) (Figure 6H).

## 4. Discussion

Growing epidemiological and experimental data support the concept of the gut–lung axis, highlighting the connection between gut microbiota and lung health [29,30,31,32,33,52]. This axis involves two-way communication between the gastrointestinal and respiratory mucosa, mediated by microbial interactions, immune responses, and metabolic byproducts, which appear to influence the development and progression of several diseases, including COVID-19 [28,36,53,54,55,56]. Nevertheless, the bacteriome profile in SARS-CoV-2-infected Brazilian patients and the impact on gut permeability and systemic inflammatory cytokines have not yet been studied. In this observational study, we investigated the gut bacteriome and permeability in COVID-19 and PCC patients and their correlation with systemic inflammatory cytokines in a cohort enrolled in the pre-vaccination period in Brazil.

Previous studies in different countries have reported significant alterations in the gut microbiome in COVID-19 patients, including decreased microbial richness and diversity, along with a predominance of opportunistic microorganisms and impaired short-chain fatty acid biosynthesis [57,58,59,60,61,62,63]. Some studies have identified a microbiota fingerprint associated with both disease severity and mortality in COVID-19, showing its potential role as a disease severity predictor in hospitalized patients [64,65]. In addition, a marked reduction in beneficial gut commensals has been documented and has been inversely correlated with pro-inflammatory cytokines and disease severity [57,58,59,60,66]. Notably, more than 20% of adult COVID-19 patients fail to recover within three months, and an imbalance in the gut microbiota persists even after SARS-CoV-2 virus negativity and respiratory symptom resolution [60,67]. Recent studies have shown intestinal dysbiosis in PCC patients and suggested a relationship between long-term sequalae, microbiota disruption, and immune dysfunction [68,69,70,71,72,73]. In the present study, we also observed significant differences in alpha and beta diversity metrics in COVID-19 and PCC patients compared to control subjects. These differences were characterized by an increase in potentially pathogenic taxa (e.g., *Enterobacteriaceae*, *Escherichia-Shigella*) and a reduction in commensal microbes (e.g., *Blautia*, *Alistipes*), consistent with previous reports. However, we were unable to identify reliable biomarkers of disease severity, likely due to the limited number of severe cases within our cohort.

Concerning systemic immunity in COVID-19, clinical studies have shown a significant rise in pro-inflammatory cytokines and chemokines (IL-1β, IL-2, IL-6, IL-8, IL-15, IFN-γ, TNF, MCP-1), characterizing cytokine release syndrome (CRS) or cytokine storm [74,75,76,77,78,79,80]. CRS may arise directly from viral-induced tissue damage or indirectly through an overactive immune response, which leads to the infiltration of immune cells into affected tissues. Although this infiltration is initially intended to contain viral spread, it ultimately causes more damage than benefits [75,76]. The excessive immune response contributes to severe manifestations like ARDS, MOF, and increased mortality [74,75,76]. Also, immune dysfunctions with impaired interaction between cellular and humoral adaptive immunity were observed in PCC patients, potentially resulting in immune dysregulation, persistent inflammation, and the clinical symptoms characteristic of this debilitating condition [25,26,27,81,82]. In our study, we detected a significant increase in plasma concentrations of IL-2, IL-6, IL-17A, and IFN-γ in SARS-CoV-2-infected patients in agreement with previous studies. Increased IL-6 levels have been consistently associated with disease severity in COVID-19 patients, as have higher concentrations of C-reactive protein, a downstream inflammatory marker [83,84,85,86]. Increased IL-17 has been observed in severe COVID-19 and correlated with lung lesions and ARDS development [74,83]. Increased IFN-γ plays a central role in promoting a highly inflammatory macrophage phenotype within the lungs of severe COVID-19 patients [20,87]. Similarly to other publications, we found some correlations between the bacteriome and inflammatory cytokines in our Brazilian cohort, suggesting that the bacteriome may influence the host’s immune response to SARS-CoV-2 [58,60,66,71].

In addition, SARS-CoV-2 infection has been associated with increased gut permeability, which may contribute to systemic inflammation and disease severity. Disruption of the gut epithelial barrier during COVID-19 and PCC can allow microbial products such as lipopolysaccharides and other endotoxins to translocate into the bloodstream, amplifying the host’s inflammatory response [37,38,39,88,89]. Elevated levels of gut permeability markers, such as zonulin, LPS-binding protein, and intestinal fatty acid-binding protein, have been detected in COVID-19 patients and correlate with worse clinical outcomes [90,91,92]. The gut–lung axis may play a critical role in COVID-19 pathophysiology, where compromised intestinal barrier function exacerbates pulmonary inflammation through the systemic circulation of pro-inflammatory mediators and favors bacterial translocation and bacteremia [91,92]. We also detected increased gut permeability in our cohort, with high plasma zonulin levels in COVID-19 and PCC patients, in addition to a strong positive correlation with pathogenic *Enterobacteriaceae* and *Escherichia-Shigella*, suggesting the involvement of a triad consisting of intestinal dysbiosis, leaky gut, and systemic inflammation in SARS-CoV-2-infected patients.

Our findings underscore the significance of the gut–lung axis and highlight the role of the gut microbiota in modulating immune responses and preserving intestinal barrier integrity. These results suggest a potential avenue for modulating immunity to SARS-CoV-2 through gut microbiota interventions. However, our study presents some limitations, including (1) the absence of stratified analyses based on disease severity and the use of antibiotics and (2) the lack of identification of circulating SARS-CoV-2 variants and quantification of short-chain fatty acids. Despite these limitations, this study presents notable strengths: (1) the inclusion of patients from the pre-vaccination period, removing vaccination as a confounding factor, and (2) the novel evaluation of microbiota, cytokines, and intestinal permeability in a Brazilian cohort—providing valuable insights into the gut–lung axis and its relevance to COVID-19 pathophysiology and potential long-term effects.

## Figures and Tables

**Table 1:** Clinical and demographic characteristics of SARS-CoV-2-infected patients and controls.

	COVID-19 (*n* = 79)	PCC (*n* = 141)	CTL (*n* = 97)
Biological sex Female/Male	46 F/33 M	88 F/53 M	83 F/14 M
Age (Years) Mean ± SD	51.8 ± 16.3	40.8 ± 13.9	43.8 ± 13.6
BMI (Kg/m^2^) Mean ± SD	28.6 ± 6.2	29.1 ± 5.4	25.2 ± 4.8
CRP (mg/dL) Mean ± SD	78.6 ± 67.0	8.4 ± 9.6	-

F: female; M: male; SD: standard deviation; BMI: body mass index; Kg/m^2^: kilograms per square meters; CRP: C-reactive protein; mg/dL: milligrams per deciliter.

## Data Availability

The 16S sequencing raw data has been deposited in the NCBI Sequence Read Archive (SRA) under BioProject PRJNA1189098.

## References

[1] Hu B., Guo H., Zhou P., Shi Z.L. (2021). Characteristics of SARS-CoV-2 and COVID-19. Nat. Rev. Microbiol..

[2] Senevirathne T.H., Wekking D., Swain J.W.R., Solinas C., De Silva P. (2024). COVID-19: From emerging variants to vaccination. Cytokine Growth Factor Rev..

[3] World Health Organization WHO COVID-19 Dashboard. https://data.who.int/dashboards/covid19/cases?n=c.

[4] Brazilian Ministry of Health Coronavirus Panel. https://covid.saude.gov.br/.

[5] Wiersinga W.J., Rhodes A., Cheng A.C., Peacock S.J., Prescott H.C. (2020). Pathophysiology, Transmission, Diagnosis, and Treatment of Coronavirus Disease 2019 (COVID-19): A Review. JAMA.

[6] Gandhi R.T., Lynch J.B., Del Rio C. (2020). Mild or Moderate Covid-19. N. Engl. J. Med..

[7] Berlin D.A., Gulick R.M., Martinez F.J. (2020). Severe Covid-19. N. Engl. J. Med..

[8] Brodin P. (2021). Immune determinants of COVID-19 disease presentation and severity. Nat. Med..

[9] Gheorghita R., Soldanescu I., Lobiuc A., Caliman Sturdza O.A., Filip R., Constantinescu-Bercu A., Dimian M., Mangul S., Covasa M. (2024). The knowns and unknowns of long COVID-19: From mechanisms to therapeutical approaches. Front. Immunol..

[10] Hurme A., Viinanen A., Teräsjärvi J., Jalkanen P., Feuth T., Löyttyniemi E., Vuorinen T., Kantele A., Oksi J., He Q. (2025). Post-COVID-19 condition in prospective inpatient and outpatient cohorts. Sci. Rep..

[11] Nalbandian A., Sehgal K., Gupta A., Madhavan M.V., McGroder C., Stevens J.S., Cook J.R., Nordvig A.S., Shalev D., Sehrawat T.S. (2021). Post-acute COVID-19 syndrome. Nat. Med..

[12] Parotto M., Gyöngyösi M., Howe K., Myatra S.N., Ranzani O., Shankar-Hari M., Herridge M.S. (2023). Post-acute sequelae of COVID-19: Understanding and addressing the burden of multisystem manifestations. Lancet Respir. Med..

[13] Santos M., Dorna M., Franco E., Geronutti J., Brizola L., Ishimoto L., Barros Y., Costa A., Breda C., Marin C. (2024). Clinical and Physiological Variables in Patients with Post-COVID-19 Condition and Persistent Fatigue. J. Clin. Med..

[14] Davis H.E., McCorkell L., Vogel J.M., Topol E.J. (2023). Long COVID: Major findings, mechanisms and recommendations. Nat. Rev. Microbiol..

[15] Oelsner E.C., Sun Y., Balte P.P., Allen N.B., Andrews H., Carson A., Cole S.A., Coresh J., Couper D., Cushman M. (2024). Epidemiologic Features of Recovery From SARS-CoV-2 Infection. JAMA Netw. Open.

[16] Soriano J.B., Murthy S., Marshall J.C., Relan P., Diaz J.V. (2022). WHO Clinical Case Definition Working Group on Post-COVID-19 Condition. A clinical case definition of post-COVID-19 condition by a Delphi consensus. Lancet Infect. Dis..

[17] Lamers M.M., Haagmans B.L. (2022). SARS-CoV-2 pathogenesis. Nat. Rev. Microbiol..

[18] Steiner S., Kratzel A., Barut G.T., Lang R.M., Aguiar Moreira E., Thomann L., Kelly J.N., Thiel V. (2024). SARS-CoV-2 biology and host interactions. Nat. Rev. Microbiol..

[19] Pozdnyakova V., Weber B., Cheng S., Ebinger J.E. (2025). Review of Immunologic Manifestations of COVID-19 Infection and Vaccination. Rheum. Dis. Clin. N. Am..

[20] Karki R., Sharma B.R., Tuladhar S., Williams E.P., Zalduondo L., Samir P., Zheng M., Sundaram B., Banoth B., Malireddi R.K.S. (2021). Synergism of TNF-α and IFN-γ Triggers Inflammatory Cell Death, Tissue Damage, and Mortality in SARS-CoV-2 Infection and Cytokine Shock Syndromes. Cell.

[21] Diamond M.S., Kanneganti T.D. (2022). Innate immunity: The first line of defense against SARS-CoV-2. Nat. Immunol..

[22] Zheng M., Karki R., Williams E.P., Yang D., Fitzpatrick E., Vogel P., Jonsson C.B., Kanneganti T.-D. (2021). TLR2 senses the SARS-CoV-2 envelope protein to produce inflammatory cytokines. Nat. Immunol..

[23] Sette A., Crotty S. (2021). Adaptive immunity to SARS-CoV-2 and COVID-19. Cell.

[24] Qi H., Liu B., Wang X., Zhang L. (2022). The humoral response and antibodies against SARS-CoV-2 infection. Nat. Immunol..

[25] Phetsouphanh C., Darley D.R., Wilson D.B., Howe A., Munier C.M.L., Patel S.K., Juno J.A., Burrell L.M., Kent S.J., Dore G.J. (2022). Immunological dysfunction persists for 8 months following initial mild-to-moderate SARS-CoV-2 infection. Nat. Immunol..

[26] Yin K., Peluso M.J., Luo X., Thomas R., Shin M.G., Neidleman J., Andrew A., Young K.C., Ma T., Hoh R. (2024). Long COVID manifests with T cell dysregulation, inflammation and an uncoordinated adaptive immune response to SARS-CoV-2. Nat. Immunol..

[27] Phetsouphanh C., Jacka B., Ballouz S., Jackson K.J.L., Wilson D.B., Manandhar B., Klemm V., Tan H.-X., Wheatley A., Aggarwal A. (2024). Improvement of immune dysregulation in individuals with long COVID at 24-months following SARS-CoV-2 infection. Nat. Commun..

[28] Oliveira G.L.V., Oliveira C.N.S., Pinzan C.F., de Salis L.V.V., Cardoso C.R.B. (2021). Microbiota Modulation of the Gut-Lung Axis in COVID-19. Front. Immunol..

[29] Budden K.F., Gellatly S.L., Wood D.L., Cooper M.A., Morrison M., Hugenholtz P., Hansbro P.M. (2017). Emerging pathogenic links between microbiota and the gut-lung axis. Nat. Rev. Microbiol..

[30] Grayson M.H., Camarda L.E., Hussain S.A., Zemple S.J., Hayward M., Lam V., Hunter D.A., Santoro J.L., Rohlfing M., Cheung D.S. (2018). Intestinal Microbiota Disruption Reduces Regulatory T Cells and Increases Respiratory Viral Infection Mortality Through Increased IFNγ Production. Front. Immunol..

[31] Dang A.T., Marsland B.J. (2019). Microbes, metabolites, and the gut-lung axis. Mucosal Immunol..

[32] Enaud R., Prevel R., Ciarlo E., Beaufils F., Wieërs G., Guery B., Delhaes L. (2020). The Gut-Lung Axis in Health and Respiratory Diseases: A Place for Inter-Organ and Inter-Kingdom Crosstalks. Front. Cell. Infect. Microbiol..

[33] Zhang D., Li S., Wang N., Tan H.Y., Zhang Z., Feng Y. (2020). The Cross-Talk Between Gut Microbiota and Lungs in Common Lung Diseases. Front. Microbiol..

[34] Stefan K.L., Kim M.V., Iwasaki A., Kasper D.L. (2020). Commensal Microbiota Modulation of Natural Resistance to Virus Infection. Cell.

[35] Ngo V.L., Lieber C.M., Kang H.J., Sakamoto K., Kuczma M., Plemper R.K., Gewirtz A.T. (2024). Intestinal microbiota programming of alveolar macrophages influences severity of respiratory viral infection. Cell Host Microbe.

[36] Zhang Y., Ma Y., Sun W., Zhou X., Wang R., Xie P., Dai L., Gao Y., Li J. (2024). Exploring gut-lung axis crosstalk in SARS-CoV-2 infection: Insights from a hACE2 mouse model. J. Med. Virol..

[37] Giron L.B., Dweep H., Yin X., Wang H., Damra M., Goldman A.R., Gorman N., Palmer C.S., Tang H.-Y., Shaikh M.W. (2021). Plasma Markers of Disrupted Gut Permeability in Severe COVID-19 Patients. Front. Immunol..

[38] Prasad R., Patton M.J., Floyd J.L., Fortmann S., DuPont M., Harbour A., Wright J., Lamendella R., Stevens B.R., Oudit G.Y. (2022). Plasma Microbiome in COVID-19 Subjects: An Indicator of Gut Barrier Defects and Dysbiosis. Int. J. Mol. Sci..

[39] Bernard-Raichon L., Venzon M., Klein J., Axelrad J.E., Zhang C., Sullivan A.P., Hussey G.A., Casanovas-Massana A., Noval M.G., Valero-Jimenez A.M. (2022). Gut microbiome dysbiosis in antibiotic-treated COVID-19 patients is associated with microbial translocation and bacteremia. Nat. Commun..

[40] Andrews S. (2010). FastQC: A Quality Control Tool for High Throughput Sequence Data.

[41] Callahan B.J., McMurdie P.J., Rosen M.J., Han A.W., Johnson A.J., Holmes S.P. (2016). DADA2: High-resolution sample inference from Illumina amplicon data. Nat. Methods.

[42] Cole J.R., Chai B., Farris R.J., Wang Q., Kulam S.A., McGarrell D.M., Garrity G.M., Tiedje J.M. (2005). The Ribosomal Database Project (RDP-II): Sequences and tools for high-throughput rRNA analysis. Nucleic Acids Res..

[43] Altschul S.F., Gish W., Miller W., Myers E.W., Lipman D.J. (1990). Basic local alignment search tool. J. Mol. Biol..

[44] Schliep K.P. (2011). phangorn: Phylogenetic analysis in R. Bioinformatics.

[45] McMurdie P.J., Holmes S. (2013). phyloseq: An R package for reproducible interactive analysis and graphics of microbiome census data. PLoS ONE.

[46] Mikkelsen V. (2023). metagMisc: Miscellaneous Functions for Metagenomic Analysis. R package version 1.0.0. 2022. https://github.com/vmikk/metagMisc.

[47] Andersen K.S., Kirkegaard R.H., Karst S.M., Albertsen M. (2018). ampvis2: An R Package to Analyse and Visualise 16S rRNA Amplicon Data. bioRxiv.

[48] Lahti L., Shetty S. (2012). Microbiome: An R Package for Microbiome Analysis. [R Package]. https://microbiome.github.io/microbiome/.

[49] Oksanen J., Simpson G.L., Blanchet F.G., Kindt R., Legendre P., Minchin P.R., O’HAra R., Solymos P., Stevens M.H.H., Szoecs E. vegan: Community Ecology Package; 2022. https://cran.r-project.org/package=vegan.

[50] Arbizu P.M. pairwiseAdonis: Pairwise Multilevel Comparison Using Adonis; 2017. https://github.com/pmartinezarbizu/pairwiseAdonis.

[51] Wickham H. (2016). ggplot2: Elegant Graphics for Data Analysis.

[52] Sencio V., Machado M.G., Trottein F. (2021). The lung-gut axis during viral respiratory infections: The impact of gut dysbiosis on secondary disease outcomes. Mucosal Immunol..

[53] Allali I., Bakri Y., Amzazi S., Ghazal H. (2021). Gut-Lung Axis in COVID-19. Interdiscip. Perspect. Infect. Dis..

[54] Yang Y., Huang W., Fan Y., Chen G.Q. (2021). Gastrointestinal Microenvironment and the Gut-Lung Axis in the Immune Responses of Severe COVID-19. Front. Mol. Biosci..

[55] Wang B., Zhang L., Wang Y., Dai T., Qin Z., Zhou F., Zhang L. (2022). Alterations in microbiota of patients with COVID-19: Potential mechanisms and therapeutic interventions. Signal Transduct. Target. Ther..

[56] Zhang F., Lau R.I., Liu Q., Su Q., Chan F.K.L., Ng S.C. (2023). Gut microbiota in COVID-19: Key microbial changes, potential mechanisms and clinical applications. Nat. Rev. Gastroenterol. Hepatol..

[57] Zuo T., Zhang F., Lui G.C.Y., Yeoh Y.K., Li A.Y.L., Zhan H., Wan Y., Chung A.C.K., Cheung C.P., Chen N. (2020). Alterations in Gut Microbiota of Patients With COVID-19 During Time of Hospitalization. Gastroenterology.

[58] Gu S., Chen Y., Wu Z., Chen Y., Gao H., Lv L., Guo F., Zhang X., Luo R., Huang C. (2020). Alterations of the Gut Microbiota in Patients With Coronavirus Disease 2019 or H1N1 Influenza. Clin. Infect. Dis..

[59] Zuo T., Liu Q., Zhang F., Lui G.C., Tso E.Y., Yeoh Y.K., Chen Z., Boon S., Chan F.K.L., Chan P. (2021). Depicting SARS-CoV-2 faecal viral activity in association with gut microbiota composition in patients with COVID-19. Gut.

[60] Yeoh Y.K., Zuo T., Lui G.C., Zhang F., Liu Q., Li A.Y., Chung A.C., Cheung C.P., Tso E.Y., Fung K.S. (2021). Gut microbiota composition reflects disease severity and dysfunctional immune responses in patients with COVID-19. Gut.

[61] Nobre J.G., Delgadinho M., Silva C., Mendes J., Mateus V., Ribeiro E., Costa D.A., Lopes M., Pedroso A.I., Trigueiros F. (2022). Gut microbiota profile of COVID-19 patients: Prognosis and risk stratification (MicroCOVID-19 study). Front. Microbiol..

[62] Shimizu K., Hirata H., Tokuhira N., Motooka D., Nakamura S., Ueda A., Tachino J., Koide M., Uchiyama A., Ogura H. (2024). Dysbiosis of gut microbiota in patients with severe COVID-19. Acute Med. Surg..

[63] Yokoyama Y., Ichiki T., Yamakawa T., Tsuji Y., Kuronuma K., Takahashi S., Narimatsu E., Katanuma A., Nakase H. (2024). Gut microbiota and metabolites in patients with COVID-19 are altered by the type of SARS-CoV-2 variant. Front. Microbiol..

[64] Bucci V., Ward D.V., Bhattarai S., Rojas-Correa M., Purkayastha A., Holler D., Da Qu M., Mitchell W.G., Yang J., Fountain S. (2023). The intestinal microbiota predicts COVID-19 severity and fatality regardless of hospital feeding method. mSystems.

[65] Zhong J., Guo L., Wang Y., Jiang X., Wang C., Xiao Y., Wang Y., Zhou F., Wu C., Chen L. (2024). Gut Microbiota Improves Prognostic Prediction in Critically Ill COVID-19 Patients Alongside Immunological and Hematological Indicators. Research.

[66] Nagata N., Takeuchi T., Masuoka H., Aoki R., Ishikane M., Iwamoto N., Sugiyama M., Suda W., Nakanishi Y., Terada-Hirashima J. (2023). Human Gut Microbiota and Its Metabolites Impact Immune Responses in COVID-19 and Its Complications. Gastroenterology.

[67] An Y., He L., Xu X., Piao M., Wang B., Liu T., Cao H. (2024). Gut microbiota in post-acute COVID-19 syndrome: Not the end of the story. Front. Microbiol..

[68] Chen Y., Gu S., Chen Y., Lu H., Shi D., Guo J., Wu W.-R., Yang Y., Li Y., Xu K.-J. (2022). Six-month follow-up of gut microbiota richness in patients with COVID-19. Gut.

[69] Liu Q., Mak J.W.Y., Su Q., Yeoh Y.K., Lui G.C., Ng S.S.S., Zhang F., Li A.Y.L., Lu W., Hui D.S.-C. (2022). Gut microbiota dynamics in a prospective cohort of patients with post-acute COVID-19 syndrome. Gut.

[70] Ferreira-Junior A.S., Borgonovi T.F., De Salis L.V.V., Leite A.Z., Dantas A.S., De Salis G.V.V., Cruz G.N.F., De Oliveira L.F.V., Gomes E., Penna A.L.B. (2022). Detection of Intestinal Dysbiosis in Post-COVID-19 Patients One to Eight Months after Acute Disease Resolution. Int. J. Environ. Res. Public. Health.

[71] Zhang F., Wan Y., Zuo T., Yeoh Y.K., Liu Q., Zhang L., Zhan H., Lu W., Xu W., Lui G.C. (2022). Prolonged Impairment of Short-Chain Fatty Acid and L-Isoleucine Biosynthesis in Gut Microbiome in Patients With COVID-19. Gastroenterology.

[72] Su Q., Lau R.I., Liu Q., Li M.K.T., Yan Mak J.W., Lu W., Lau I.S., Lau L.H., Yeung G.T., Cheung C.P. (2024). The gut microbiome associates with phenotypic manifestations of post-acute COVID-19 syndrome. Cell Host Microbe.

[73] Blankestijn J.M., Baalbaki N., Beijers R.J.H.C.G., Cornelissen M.E.B., Wiersinga W.J., Abdel-Aziz M.I., der Zee A.H.M.-V. (2025). P4O2 Consortium. Exploring Heterogeneity of Fecal Microbiome in Long COVID Patients at 3 to 6 Months After Infection. Int. J. Mol. Sci..

[74] Darif D., Hammi I., Kihel A., El Idrissi Saik I., Guessous F., Akarid K. (2021). The pro-inflammatory cytokines in COVID-19 pathogenesis: What goes wrong?. Microb. Pathog..

[75] Zanza C., Romenskaya T., Manetti A.C., Franceschi F., La Russa R., Bertozzi G., Maiese A., Savioli G., Volonnino G., Longhitano Y. (2022). Cytokine Storm in COVID-19: Immunopathogenesis and Therapy. Medicina.

[76] Deng X., Tang K., Wang Z., He S., Luo Z. (2024). Impacts of Inflammatory Cytokines Variants on Systemic Inflammatory Profile and COVID-19 Severity. J. Epidemiol. Glob. Health.

[77] Islam F., Habib S., Badruddza K., Rahman M., Islam M.R., Sultana S., Nessa A. (2024). The Association of Cytokines IL-2, IL-6, TNF-α, IFN-γ, and IL-10 With the Disease Severity of COVID-19: A Study From Bangladesh. Cureus.

[78] Safont G., Villar-Hernández R., Smalchuk D., Stojanovic Z., Marín A., Lacoma A., Pérez-Cano C., López-Martínez A., Molina-Moya B., Solis A.J. (2024). Measurement of IFN-γ and IL-2 for the assessment of the cellular immunity against SARS-CoV-2. Sci. Rep..

[79] Qin C., Zhou L., Hu Z., Zhang S., Yang S., Tao Y., Xie C., Ma K., Shang K., Wang W. (2020). Dysregulation of Immune Response in Patients With Coronavirus 2019 (COVID-19) in Wuhan, China. Clin. Infect. Dis..

[80] Hu B., Huang S., Yin L. (2021). The cytokine storm and COVID-19. J. Med. Virol..

[81] Kervevan J., Staropoli I., Slama D., Jeger-Madiot R., Donnadieu F., Planas D., Pietri M.-P., Loghmari-Bouchneb W., Tanah M.A., Robinot R. (2023). Divergent adaptive immune responses define two types of long COVID. Front. Immunol..

[82] Adhikari A., Maddumage J., Eriksson E.M., Annesley S.J., Lawson V.A., Bryant V.L., Gras S. (2024). Beyond acute infection: Mechanisms underlying post-acute sequelae of COVID-19 (PASC). Med. J. Aust..

[83] Lucas C., Wong P., Klein J., Castro T.B.R., Silva J., Sundaram M., Ellingson M.K., Mao T., Oh J.E., Israelow B. (2020). Longitudinal analyses reveal immunological misfiring in severe COVID-19. Nature.

[84] Mahmood S.B.Z., Majid H., Arshad A., Zaib-Un-Nisa, Niazali N., Kazi K., Aslam A., Ahmed S., Jamil B., Jafri L. (2023). Interleukin-6 (IL-6) as a Predictor of Clinical Outcomes in Patients with COVID-19. Clin. Lab..

[85] Herold T., Jurinovic V., Arnreich C., Lipworth B.J., Hellmuth J.C., von Bergwelt-Baildon M., Klein M., Weinberger T. (2020). Elevated levels of IL-6 and CRP predict the need for mechanical ventilation in COVID-19. J. Allergy Clin. Immunol..

[86] Chen X., Zhao B., Qu Y., Chen Y., Xiong J., Feng Y., Men D., Huang Q., Liu Y., Yang B. (2020). Detectable Serum Severe Acute Respiratory Syndrome Coronavirus 2 Viral Load (RNAemia) Is Closely Correlated With Drastically Elevated Interleukin 6 Level in Critically Ill Patients With Coronavirus Disease 2019. Clin. Infect. Dis..

[87] Zhang F., Mears J.R., Shakib L., Beynor J.I., Shanaj S., Korsunsky I., Nathan A., Donlin L.T., Raychaudhuri S. (2021). IFN-γ and TNF-α drive a CXCL10+ CCL2+ macrophage phenotype expanded in severe COVID-19 lungs and inflammatory diseases with tissue inflammation. Genome Med..

[88] Mouchati C., Durieux J.C., Zisis S.N., Labbato D., Rodgers M.A., Ailstock K., Reinert B.L., Funderburg N.T., McComsey G.A. (2023). Increase in gut permeability and oxidized ldl is associated with post-acute sequelae of SARS-CoV-2. Front. Immunol..

[89] Gallo A., Murace C.A., Corbo M.M., Sarlo F., De Ninno G., Baroni S., Fancello G., Masucci L., Covino M., Tosato M. (2025). Gemelli against COVID-19 Post-Acute Care Team. Intestinal Inflammation and Permeability in Patients Recovered from SARS-CoV-2 Infection. Dig. Dis..

[90] Oliva A., Miele M.C., Di Timoteo F., De Angelis M., Mauro V., Aronica R., Al Ismail D., Ceccarelli G., Pinacchio C., d’Ettorre G. (2021). Persistent Systemic Microbial Translocation and Intestinal Damage During Coronavirus Disease-19. Front. Immunol..

[91] Oliva A., Cammisotto V., Cangemi R., Ferro D., Miele M.C., De Angelis M., Cancelli F., Pignatelli P., Venditti M., Pugliese F. (2021). Low-Grade Endotoxemia and Thrombosis in COVID-19. Clin. Transl. Gastroenterol..

[92] Palomino-Kobayashi L.A., Ymaña B., Ruiz J., Mayanga-Herrera A., Ugarte-Gil M.F., Pons M.J. (2022). Zonulin, a marker of gut permeability, is associated with mortality in a cohort of hospitalised peruvian COVID-19 patients. Front. Cell. Infect. Microbiol..

